# An adsorbent monolith device to augment the removal of uraemic toxins during haemodialysis

**DOI:** 10.1007/s10856-014-5173-9

**Published:** 2014-02-27

**Authors:** Susan R. Sandeman, Carol A. Howell, Gary J. Phillips, Yishan Zheng, Guy Standen, Robert Pletzenauer, Andrew Davenport, Kolitha Basnayake, Owen Boyd, Stephen Holt, Sergey V. Mikhalovsky

**Affiliations:** 1 Biomaterials and Medical Devices Research Group, School of Pharmacy and Biomolecular Sciences, University of Brighton, Huxley Building, Lewes Road, Brighton, East Sussex BN2 4GJ UK; 2 UCL Centre for Nephrology, Royal Free Hospital, London, NW3 2PF UK; 3 Brighton and Sussex University Hospitals NHS Trust, The Royal Sussex County Hospital, Eastern Road, Brighton, BN2 5BE UK; 4 School of Engineering, Nazarbayev University, 53 Kabanbay batyr Avenue, Astana, 010000 Kazakhstan

## Abstract

Adsorbents designed with porosity which allows the removal of protein bound and high molecular weight uraemic toxins may improve the effectiveness of haemodialysis treatment of chronic kidney disease (CKD). A nanoporous activated carbon monolith prototype designed for direct blood contact was first assessed for its capacity to remove albumin bound marker toxins indoxyl sulphate (IS), *p*-cresyl sulphate (*p*-CS) and high molecular weight cytokine interleukin-6 in spiked healthy donor studies. Haemodialysis patient blood samples were then used to measure the presence of these markers in pre- and post-dialysis blood and their removal by adsorbent recirculation of post-dialysis blood samples. Nanopores (20–100 nm) were necessary for marker uraemic toxin removal during in vitro studies. Limited removal of IS and *p*-CS occurred during haemodialysis, whereas almost complete removal occurred following perfusion through the carbon monoliths suggesting a key role for such adsorbent therapies in CKD patient care.

## Introduction

Haemodialysis is a well-established, life sustaining therapy for patients who progress to chronic kidney disease stage 5 (CKD5d) and do not receive a transplant. However morbidity and mortality rates, particularly those associated with cardiovascular disease (CVD) remain high despite improved disease management and advances in high flux and haemodiafiltration technology [1, 2]. The increase in prevalence, rising costs and poor prognosis associated with CKD5d are well established. Over 2 million CKD5 patients are on dialysis worldwide with a prevalence rate rising by 5–8 % each year in developed countries in line with the increasing incidence of diabetes, hypertension and obesity [3–5]. Whilst less than 0.1 % of the European population receive haemodialysis treatment, costs total 2 % of the healthcare budget [6]. Despite the relatively high cost of dialysis patient survival remains poor. Five year survival data from the UK Renal Registry showed survival for dialysis patients was marginally better than that for ovarian cancer, but worse than that for colon cancer [7].

The pathophysiology linking CKD and CVD progression is multi-faceted but has a clear association with chronic exposure of the endothelium to an inflammatory and pro-atherogenic environment linked to chronic cytokine expression and the action of protein bound and high molecular weight uraemic toxins [8–15]. Current haemodialysis systems predominantly remove small, water soluble metabolites and some low molecular weight proteins through diffusion and convection but fail to remove significant amounts those uraemic toxins whose retention might be most damaging. The current study used well characterised high molecular weight cytokine interleukin 6 (IL-6) and protein bound indoxyl sulphate (IS) and *p*-cresyl sulphate (*p*-CS) as marker molecules for this group. Raised IL-6 is a predictive marker for all cause and CVD related morbidity and mortality in CKD patients [16]. IS (213 Da) and *p*-CS (188 Da)are small molecules derived from gut bacterial metabolism of dietary proteins but are predominantly non-covalently bound in the plasma to 66 kDa albumin and are therefore poorly removed by haemodialysis. Both are instigators of sustained oxidative stress with subsequent, adverse effects on cardiovascular, renal and bone physiology and are considered prime targets for removal through sorbent based strategies [17–23].

Examples of sorbent containing extracorporeal devices include the molecular adsorbent regenerating system (MARS™), fractionated plasma separation and adsorption system (Prometheus™) and Hemocleanse SSR [24, 25]. MARS and Prometheus have both been designed for the treatment of liver failure where HD alone is ineffective in bridging to liver regeneration or transplant. MARS contains a microporous activated carbon column and works by dialysing patient blood across an albumin impermeable high flux membrane into an albumin rich dialysate. The dialysate is then regenerated by filtration through a low flux dialysis membrane, a microporous activated carbon column and an anion exchange resin column. The Prometheus system contains two adsorber cartridges and takes a different approach in which albumin and toxins both pass across an albumin permeable, 250 kDa cut-off membrane and albumin bound toxins are then removed in a secondary circuit by filtration through a neutral resin adsorber followed by an anion exchanger. The water soluble toxins are removed by blood filtration through a second high flux dialyzer. The Hemocleanse device also contained activated microporous carbon, and was used in the treatment of acute hepatic decompensation. However none of these devices have substantially improved patient outcomes compared to standard supportive medical management.

Haemoperfusion systems using microporous activated carbon were tested for the treatment of kidney failure more than 40 years ago and showed significant efficacy but technical problems with release of fine carbon particles, clotting, electrolyte imbalance and platelet loss limited further development [26–28]. To improve haemocompatibility most activated carbon adsorbents in current clinical use tend to be coated with cellulose or hydrogel/heparin co-polymers [29]. Unfortunately, such coatings also limit adsorptive capacity for high molecular weight molecules and the only carbon haemoadsorbents used clinically in the European Union for direct blood contact are those for the treatment of severe drug overdose [30]. Recent advances in carbon technology have allowed the development of a novel phenolic resin derived activated carbon, which can be used uncoated for direct contact with blood and has a tailored nanoporous structure allowing adsorption of larger biological toxins above 50 kDa [31–33]. A three dimensional extruded carbon monolithic block can be made with straight transport channels along its length so that adsorption occurs by direct blood contact. We have previously reported that these activated carbons do not activate complement or adsorb clotting factors to alter intrinsic or extrinsic clotting cascades [31, 34]. This study was a proof of concept study designed firstly to measure the impact of monolith porosity on the removal of selected marker uraemic toxins using in vitro methods. Following demonstration of marker molecule removal, a pilot study using clinical haemodialysis patient blood samples was then undertaken to establish whether these representative uraemic toxins remain in blood after standard haemodialysis treatment and can then be removed following adsorbent monolith haemoperfusion.

## Materials and methods

### Material synthesis and physical characterisation of porosity

Monolith adsorbents were synthesised by MAST Carbon International Ltd (Basingstoke, UK).The monoliths used were 0.7 cm wide and 10 cm long and contained 32 channels running along the length of the monolith, each with a width of 0.5 mm. Monoliths were formed by mixing and curing varying formulations of Novalac resin, ethylene glycol and hexamine. The resulting resin cake was crushed, dried or washed to remove excess pore former, milled to form a dough and then extruded. The resulting monoliths were carbonised and activated in carbon dioxide at temperatures of up to 900 °C with the nanoporosity controlled primarily by initial resin pore-former composition. The porosity of the resultant materials was characterised by scanning electron microscopy (SEM) using a Sigma field emission gun (FEG)-SEM microscope (Carl Zeiss NTS, Cambridge, UK) and by porosimetry using an Autosorb gas sorption analyser and an automatic PoreMaster^**®**^ mercury intrusion porosimeter (Quantachrome Instruments Ltd, Hook, UK). Calculations relating to surface area, pore volume and pore size distribution were carried out using Quantachrome software.

### In vitro studies to measure adsorption of IS, *p*-CS and IL-6 by monoliths with varying porosity

The ability of adsorbent monoliths to remove marker uraemic toxins was measured using healthy donor blood spiked with *p*-CS (250 μM), IS (125 μM) and IL-6 (1,000 pg/ml). A continuously circulating system was used to compare uraemic toxin removal from plasma and blood by monoliths of varying porosity. A 20 ml reservoir volume of spiked blood was circulated through each monolith pre-wetted with phosphate buffered saline (PBS) at a rate of 5 ml/min and samples were taken at timed intervals. Blood samples were centrifuged at 3,500 rpm for 15 min and plasma was removed. All samples were stored at −20 °C prior to analysis.

### HPLC analysis of IS and *p*-CS adsorption

The HPLC method was adapted from that described by de Loor et al. [35] using competitive albumin binding with sodium octanoate to measure total *p*-CS and IS in plasma. Plasma (200 μl) was incubated with 250 μl of sodium octanoate (0.24 μM) for 30 min. Internal standard (naphthalene sulphonic acid, 0.5 mM, 20 μl) was added. Samples were mixed with 2 ml of ice cold acetone to precipitate proteins and centrifuged for 10 min at 4,000 rpm. Supernatant was removed, mixed with 2 ml ice cold dichloromethane and centrifuged for 10 min at 4,000 rpm. The top aqueous phase was removed and 40 μl of 1 M HCl was added to stabilise the solution. Peak separation was achieved using a Fortis C_18_ (150 mm × 4.6 mm OD, 3 μM) column fitted with a 0.5 μm fritted Krudkatcher pre-cartridge. The column was maintained at 25 °C. Shell vials containing the samples, within spring-tip inserts, were placed in a Waters 717 plus autosampler at 15 °C which was set for a 10 μl injection, 20 min run time and 10 min report delay. A Perkin Elmer Series 200 quaternary gradient pump was programmed to deliver the mobile phases [A (%) = 0.2 % trifluoroacetic acid (TFA) in water, B (%) = 0.2 % TFA in acetonitrile as follows: 85/15 (hold 5 min)–80/20 (5 min)–0/100 (2 min, hold 3 min) at 0.6 ml/min, then 85/15 (2 min) at 1.0 ml/min]. A Varian Prostar 363 fluorescent detector was employed with a time programme. Dual detection wavelengths were used. IS and internal standard were detected initially at excitation and emission wavelengths of 280 and 360 nm, respectively. Detection of *p*-CS was accomplished at excitation and emission wavelengths of 260 and 296 nm, respectively. Peaks were visualised and integrated using Totalchrom software via a PE Nelson 900 Series (A–D) interface. Limits of detection (LOD) and quantification (LOQ) were calculated using the following equations.

$$LOD = \frac{3.3 \times \sigma }{S} ,$$

$$LOQ = \frac{10 \times \sigma }{S},$$

where S is the slope of the calibration curve and *σ* is the standard deviation of the response based on the calibration curve.

### ELISA analysis of IL-6 adsorption

Plasma samples from the adsorption studies underwent a 1 in 25 dilution in assay diluent and were analysed by ELISA according to manufacturer’s instructions (BD Biosciences, Oxford, UK). Plasma samples from the clinical sampling study were analysed by ELISA for IL-6 content without undergoing further dilution.

### Pilot study using haemodialysis patient blood samples to assess small prototype device efficacy

A small proof of concept study was conducted using haemodialysis patient blood samples. Ethical approval for the study was obtained from the National Research Ethics Committee for the South East Coast—Brighton and Sussex REC reference 11/H1111/7. Eleven patients receiving haemodialysis at the Sussex Kidney Unit were recruited to the study using an approved informed consent protocol. The patients underwent a standard 4 h, thrice weekly haemodialysis regime, using a high flux FX-10 polysulfone haemodialyser. Blood was drawn before and after haemodialysis for measurement of urea, creatinine, calcium, total protein, albumin, haemoglobin, leukocyte, erythrocyte, platelet count, IS, *p*-CS and IL-6. An additional 20 ml of post-dialysis patient blood was collected into heparinised tubes (16 IU/ml blood) and perfused through a nanoporous carbon monolith in a continuously circulating system for 90 min. Blood in this system was analysed at 0, 30, 60 and 90 min to assess removal of IS, *p*-CS, IL-6 and fibrinogen following perfusion through the monoliths over time. After 90 min blood was again analysed for changes in urea and other parameters listed above following monolith recirculation. Plasma was isolated by centrifugation at 3,500 rpm for 15 min and was stored at −20 °C prior to analysis by HPLC, ELISA and coagulometry. Changes in fibrinogen concentration were measured using a START 4 coagulometer and Fibri-prest kit using a 1:20 dilution of plasma samples (Diagnostica Stago, Theale, UK).

### Statistics

Data from in vitro studies was analysed for statistical significance using Student’s *t* test comparing monolith filtration versus control groups at 60 min. Data from the pilot study was analysed for statistical significance using Student’s *t* test comparing paired data before and after haemodialysis and before and after monolith filtration. Data is expressed as the mean value ± the standard error of the mean (s.e.m).

## Results

### Characterisation of adsorbent porosity

**Fig. 1:**
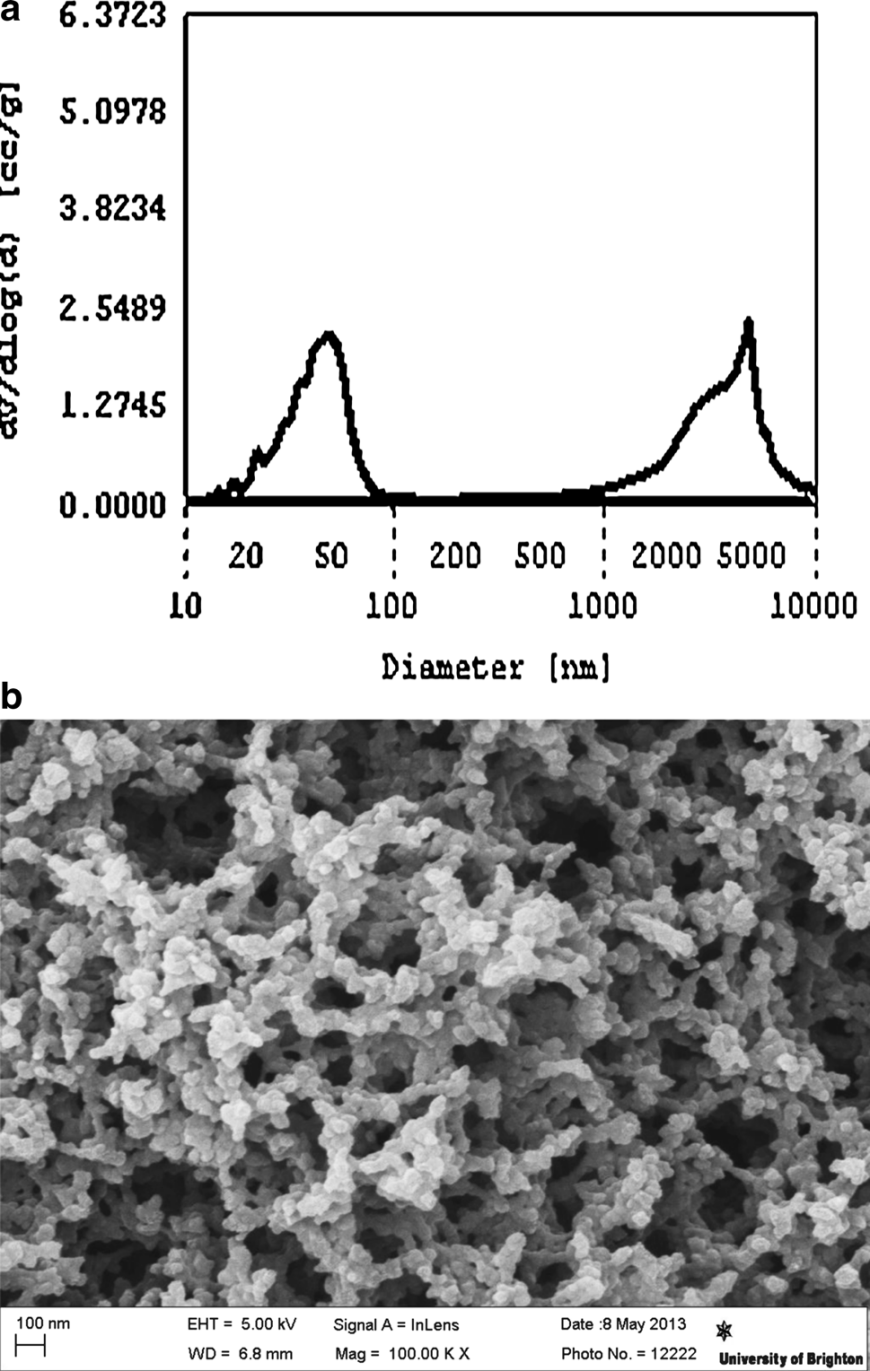
Characterisation of nanoporous monolith porosity was carried out using PoreMaster mercury intrusion porosimetry and Sigma field emission gun-scanning electron microscopy (FEG-SEM) **a** Quantachrome data reduction software was used to calculate a mercury pore size distribution plot showing nanoporous domains of 2–100 nm within the prototype carbon monoliths. The* second peak* is produced by interparticulate spaces which are 1–10 microns in diameter. **b** An SEM micrograph of a transverse section of the nanoporous activated carbon monolith showed internal nanoporous domains (magnification ×100,000)

The pore size distribution plots derived from mercury porosimetry showed differences in the porosity of the monoliths made of different resin and pore former combinations. Two types of carbon monolith were used for the in vitro study. The nanoporous monoliths had pores in the mesoporous to small macroporous range with a diameter of 2–100 nm (Fig. 1a). The microporous activated carbon monoliths had pores of less than 2 nm only. Nitrogen gas porosimetry data analysis indicated a surface area of 852 m^2^/g and a pore volume of 1.14 cc/g for the mesoporous monoliths and a surface area of 782 m^2^/g and pore volume of 0.51 cc/g for the microporous monoliths. Nanoporous domains were visible on the high magnification scanning electron micrographs taken of transverse sections of the nanoporous activated carbon monoliths (Fig. 1b).

### In vitro studies of IL-6, IS and *p*-CS adsorption by monoliths with varying porosity

**Fig. 2:**
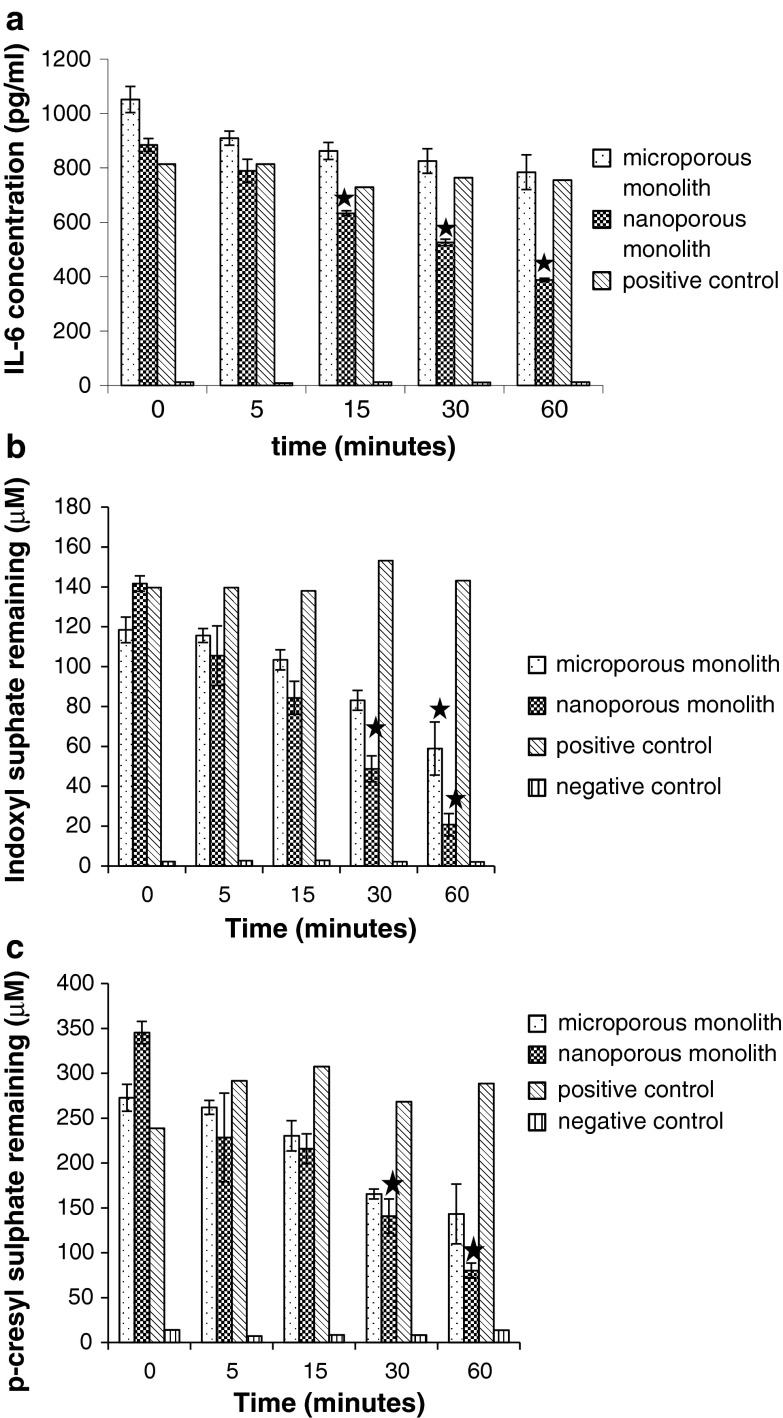
**a** IL-6, **b** indoxyl sulphate and **c** *p*-cresyl sulphate remaining following filtration of spiked fresh human blood through nanoporous monoliths, microporous monoliths and spiked and non-spiked controls (*n* = 3, mean ± s.e.m.). *Filled star* *P* < 0.01 for concentration of IL-6, IS, *p*-CS remaining following monolith filtration compared to the positive control of spiked blood circulated without a monolith at each time point

The in vitro monolith recirculation studies revealed a significant reduction in IL-6 concentration after 15 min of continuous filtration through the nanoporous monoliths (Fig. 2a, *P* < 0.01). IL-6 concentration was reduced from 900 to 400 pg/ml over the 60 min filtration cycle. In contrast, filtration through the microporous monoliths produced little reduction in the concentration of IL-6. Some removal of IS and *p*-CS occurred following continuous perfusion of spiked whole blood through the microporous carbon monoliths. The concentration of IS and *p*-CS were reduced from 120 to 60 μM and from 275 to 150 μM respectively. However, the reduction in concentration was greatest following perfusion through the nanoporous monoliths. IS concentration was reduced from 140 to 20 μM and *p*-CS was reduced from 350 to 75 μM (Fig. 2b, c). Only the nanoporous monoliths removed significant amounts of the high molecular weight cytokine IL-6. The albumin bound uraemic toxins IS and PCS were removed to some extent by microporous carbons but removal was much more effective following perfusion through the nanoporous carbon monoliths. The nanoporous monoliths were therefore used to conduct the pilot study using post haemodialysis blood samples.

### Pilot study using haemodialysis patient blood samples to assess small prototype device efficacy

**Fig. 3:**
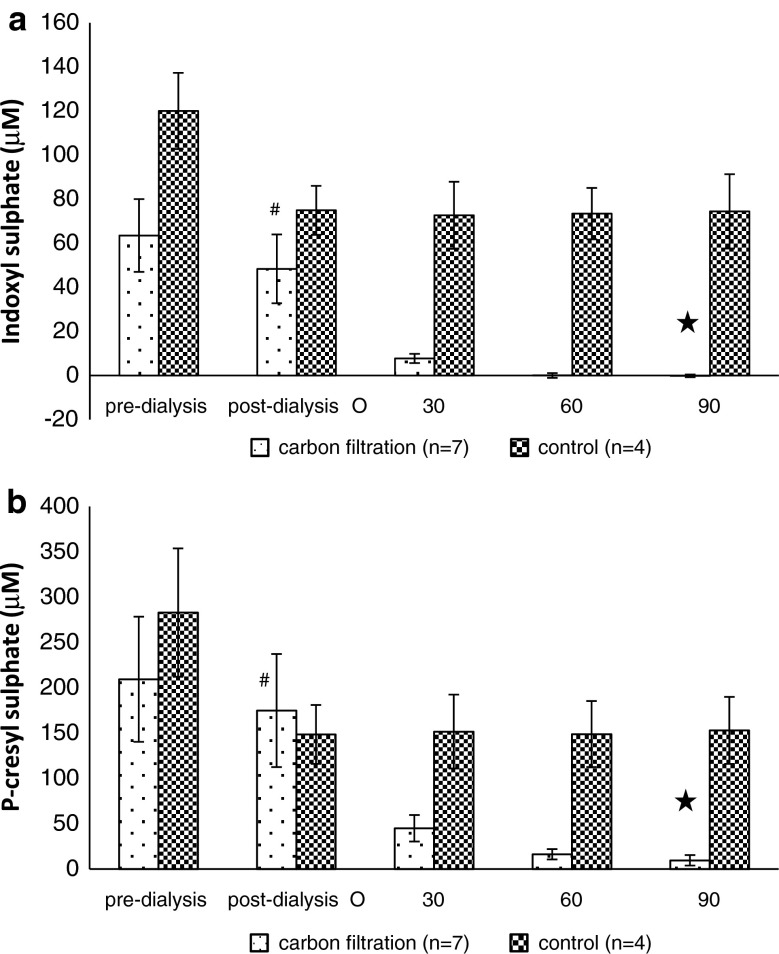
The concentration of **a** indoxyl sulphate (IS) and **b** *p*-cresyl sulphate (*p*-CS) remaining in dialysis patient blood samples pre-dialysis, post-dialysis and then after 30, 60 and 90 min filtration through the nanoporous carbon monoliths (*n* = 7) or tubing only controls (*n* = 4).Monolith filtration was carried out on 20 ml blood samples taken from patients post-dialysis. ^#^ *P* > 0.01 for pre-dialysis versus post dialysis levels of IS and *p*-CS. *Filled star P* < 0.01 for pre-dialysis compared to post-monolith filtration levels of IS and *P* = 0.01 for pre-dialysis compared to post-monolith filtration levels of *p*-CS

High concentrations of IS and *p*-CS were detected in all haemodialysis patient blood samples prior to dialysis. An insignificant reduction in IS and *p*-CS occurred following standard high flux haemodialysis (Fig. 3a, b) (*P* > 0.01). However, after 30 min of continuous perfusion of the post high flux haemodialysis patient blood samples through the nanoporous carbon monoliths the concentration of both had decreased to negligible levels (Fig. 3) (*P* < 0.01).Raised levels of IL-6 were present in only one of the monolith filtration group blood samples (patient 3, Table 1) and one of the control group samples using dialysis patient blood in a sham circuit without monolith (patient 10, Table 1). The IL-6 concentration was reduced by 50 % following carbon monolith filtration but was not reduced in the control group sample (Table 1).

**Table 1:** Measurement of IL-6 concentration (pg/ml) in blood samples taken from haemodialysis patients pre-dialysis, post dialysis and after 90 min perfusion of post-dialysis patient blood samples through a nanoporous carbon monolith (patient 1–7) or the no monolith control (patient 8–10). Significantly raised levels of IL-6 were detected in only one of the monolith (patient 3) and 1 of the control group samples (patient 10)

Patient	1	2	3	4	5	6	7	8	9	10
Pre-dialysis IL-6 (pg/ml)	<10	<10	72	<10	<10	<10	<10	14	0	421
Post dialysis IL-6 (pg/ml)	<10	<10	125	0	<10	<10	<10	<10	0	435
Post monolith or control IL-6 (pg/ml)	0	<10	53	0	0	0	0	<10	0	431

**Fig. 4:**
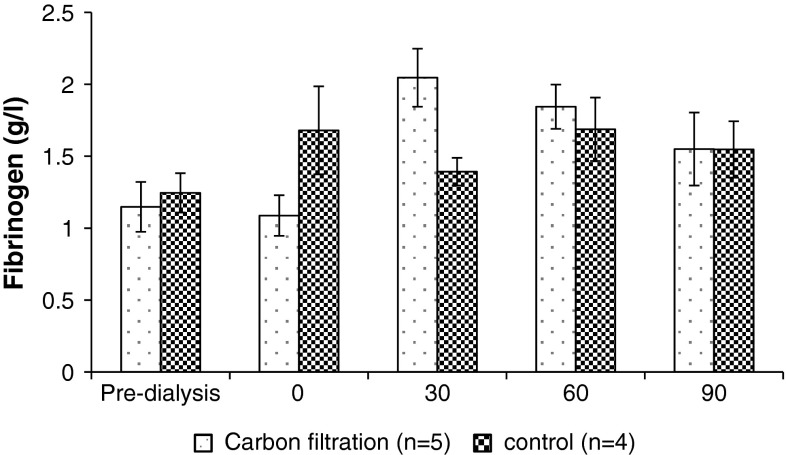
The concentration of fibrinogen measured in dialysis patient blood samples pre-dialysis, post-dialysis and after 30, 60 and 90 min filtration through the nanoporous carbon monoliths (*n* = 5) or controls (*n* = 4). *P* > 0.01 for monolith filtration versus control sample concentration of fibrinogen

Changes in blood biochemistry pre-dialysis, post-dialysis, post monolith filtration and following filtration through the system without a monolith in place are shown in Table 2. No significant difference in post monolith perfusion levels occurred when compared to control levels for all of the markers except for creatinine and total protein. Creatinine levels dropped from a mean value of 647 ± 190 μM to 256 ± 61 μM post haemodialysis. A significant reduction in creatinine levels to 37 ± 16 μM occurred following monolith filtration (*P* < 0.01) in contrast to the control group where levels remained unchanged (Table 2). The total protein levels dropped to just below normal range when compared to the control group (*P* < 0.01). The platelet count was reduced in both the monolith perfusion and control groups (*P* > 0.01) indicating that the reduction occurred as a result of blood perfusion through an extracorporeal circuit but not as a direct result of monolith contact (Table 2). No significant difference in fibrinogen concentration was observed in the carbon monolith filtration samples when compared to the controls (*P* < 0.01, Fig. 4).

**Table 2:** Changes in key blood parameters following nanoporous monolith filtration of post-haemodialysis patient blood samples compared to the no monolith controls

Monolith (*n* = 7, mean ± SD) control (*n* = 4, mean ± SD)							
	Normal range	Pre-dialysis	Post dialysis	Post monolith filtration	Pre-dialysis	Post-dialysis	Post control filtration
Urea	1.7–8.3 mmol/L	20.8 ± 7.6	6.5 ± 2.7	4.6 ± 1.8	18.5 ± 4	6.25 ± 1.2	6.65 ± 2
Creatinine	62–106 μmol/L	647 ± 190	255.6 ± 61	37 ± 16^a^	928 ± 247	373 ± 112	373 ± 112
Adjusted calcium	2.15–2.55 mmol/L	2.27 ± .2	2.4 ± .3	1.98 ± .3	2.2 ± .09	1.9 ± .05	1.9 ± .05
Total protein	66–87 g/L	65.7 ± 6.5	75 ± 4	56 ± 36^a^	65.75 ± 2.8	68.25 ± 2.6	68.2 ± 5.5
Albumin	34–48 g/L	39.3 ± 4	45.6 ± 4	34.7 ± 2.9	40.5 ± 1.73	41 ± 1.4	39.5 ± 2.6
HB	13.5–18 g/dL	11.6 ± .7	12.8 ± .55	9.9 ± 2.03	10.9 ± 1.2	11 ± 1	11.8 ± 4.3
WBC	4–11 × 10*9/L	73 ± 164	7 ± 2.9	7.2 ± 2.9	6.4 ± 1.6	6.35 ± 1	5.8 ± 2.4
Platelet	150–450 × 10*9/L	188 ± 96	203.7 ± 1.9	142.9 ± 108	195 ± 38	185 ± 11	116 ± 44
RBC	4.5–6.5 × 10*12/L	3.8 ± .5	4.2 ± .4	3.2 ± 0.65	3.4 ± .2	3.5 ± 2	3.6 ± 1.3
HCT	0.4–.54 fL	.35 ± .03	.38 ± .02	.3 ± .06	.3 ± .04	.34 ± .03	.34 ± .1

^a^Only creatinine and total protein were reduced to below normal levels compared to the no monolith controls

## Discusssion

Protein bound, middle and high molecular weight uraemic toxins accumulate during the progression of CKD and are poorly removed by current haemodialysis treatments. Our study has demonstrated that activated carbon monoliths with a large surface area for adsorption can be selectively tailored to contain nanoporous domains of up to 100 nm which are capable of adsorbing high molecular weight and protein bound uraemic toxins. In this way the activated carbon used here differs by the addition of nanopores from the microporous ‘charcoals’ used in previous extracorporeal studies. Our nanoporous monoliths are derived from synthetic precursors allowing a more consistent product profile and removing the propensity for fine carbon particle release. The monolith structure itself is also unique in an extracorporeal device and allows direct blood flow along straight channels within an extruded carbon block. Although a higher surface area for adsorption per gram of carbon could be gained from a packed carbon bead system the monolithic structure allows blood to flow without a significant pressure drop across the system. We have previously reported in vitro studies which showed improved biocompatibility of these nanoporous monoliths, observing that complement was not activated, and similarly clotting factors were not consumed [31, 34].

We sought first to establish what porous characteristics were necessary to ensure adsorption of high molecular weight cytokine IL-6, and protein bound *p*-CS and IS from spiked human blood. Our in vitro study confirmed the importance of specifically tailored nanoporous domains of 2–100 nm for the removal of high molecular weight molecules such as IL-6 and the complex mechanisms involved in carbon adsorbent binding of protein bound molecules. The monoliths with pores of up to 100 nm targeted both high molecular weight and protein bound species unlike the monoliths made from standard microporous carbons which showed negligible removal of IL-6. Some removal of the protein bound IS and *p*-CS occurred but adsorption was much less than that achieved by the nanoporous monoliths.

In the second part of the study, using pre and post haemodialysis patient blood samples, IS and *p*-CS were detected in high concentrations both pre- and post-haemodialysis and were then reduced to negligible levels following perfusion through the carbon monoliths. Blood was pumped directly through the nanoporous monoliths without causing additional reduction in platelet count or significant removal of albumin when compared to the control circuits, although an overall drop in platelet count occurred in both groups. IL-6 levels were raised in 2 of the 10 clinical samples studied indicating that a more ubiquitous high molecular weight marker is necessary to assess sorbent efficacy in a small sample pilot study. Further work is necessary to develop and assess a scaled up device that can be used in conjunction with a dialysis circuit. Although, preliminary data suggests a sustained capacity for protein bound uraemic toxin removal it should be noted that not all putative uraemic solutes are suitable for removal by activated carbon adsorption. Molecules with aromatic rings and a propensity for hydrophobic interactions are adsorbed but electrolytes and highly water soluble molecules such as urea are not removed to any significant extent as demonstrated in this and in a previous in vitro study [32].

It is interesting to note that during the in vitro studies significant IL-6 monolith adsorption required the presence of larger nanopores. In contrast some removal of the albumin bound IS and *p*-CS occurred in the microporous monoliths with pores of less than 2 nm only. IL-6 is a 185 amino-acid, pleiotropic protein with a molecular weight range of 21–26 kDa and a tertiary structure made up of four alpha helices [36]. The structural dimensions of IL-6 are 5 × 5 × 12.2 nm resulting in no significant adsorption by microporous carbon. Similarly human serum albumin is a globular protein with approximate dimensions of 8 × 8 × 3 nm [37] indicating that adsorption of IS and *p*-CS as albumin bound complexes by microporous carbons should not occur since the complex will not fit within the 2 nm wide porous domains. The normal concentration range of albumin in plasma is around 500–700 μM. We observed a mean IS concentration of 49 ± 16 μM and a mean *p*-CS concentration of 174 ± 17 μM in post dialysis patient samples. These molecules compete for the same albumin binding site so that a much larger drop in albumin would be expected if the whole albumin-IS/*p*-CS complex was simply removed by carbon monolith adsorption, assuming a binding ratio of 1:1. Albumin concentration in the haemodialysis patient blood samples dropped following monolith filtration but remained in the normal range suggesting some adsorption by the monolith but not enough to explain the almost complete removal of IS and *p*-CS. The results of our study suggest that removal is more likely to occur through disruption of the non-covalent bond between albumin and these molecules. The pore walls of activated carbon have high adsorption affinity towards aromatic substances, such as *p*-CS and IS, facilitating dissociation of their complexes with albumin.

The rapid reduction in IS and PCS concentration in the post-dialysis blood samples suggests different binding dynamics to that of the in vitro spiked studies using healthy donor blood. CKD is associated with changes in the concentration, tertiary structure and binding capacity of albumin. Albumin contains a large number of thiol groups and acts as a key circulating anti-oxidant through the formation of disulphide bonds with excess circulating free radicals [38, 39]. Chronic exposure to oxidative stress increases the population of highly oxidised forms of albumin inducing conformational changes and reducing binding and transport capacity for molecules such as IS and *p*-CS. The results suggest a greater propensity for carbon adsorption of albumin bound molecules from the haemodialysis patient blood samples due to decreased albumin binding capacity. However, further work is necessary to highlight the specific nature of molecular binding to the carbon surface and changes in the interaction of protein bound toxins with albumin and nanoporous activated carbons in patients with CKD5d.

A number of issues surround the introduction of an adsorbent device into clinical practice including the potential removal of beneficial substances and the capacity of the device to maintain sufficient clearance of uraemic toxins. Previous publications have indicated limited rebound release of *p*-CS from tissue into the vascular space 30 min after haemodialysis [40]. Rebound of small water soluble molecules urea and potassium is known to occur in CKD in the first hour following haemodialysis and rebound of cytokines in sepsis associated acute kidney injury occurs where removal by haemofiltration is matched by further production and tissue release back into the blood plasma so that no overall drop in cytokine levels is observed [41]. In the case of inflammatory mediators it may not be beneficial to clear them completely from the blood but only to remove the peak concentrations so that suppression of excessive inflammatory disregulation occurs. The non-specificity of activated carbon adsorption is preferable to single target therapies in view of the spectrum of uraemic toxins which accumulate in CKD5d. Although there may be some removal of beneficial molecules in the equivalent molecular weight range replacement is possible through normal homeostatic mechanisms. Adsorption tends to cap only the peak concentrations of dominant plasma proteins such as albumin and fibrinogen but significantly reduces the pathophysiologically high levels of uraemic toxins such as IS and *p*-CS where homeostatic regulation through renal excretion has failed. In the current study no significant reduction in albumin and fibrinogen levels occurred in the monolith filtration samples compared to the controls, although the concentration of fibrinogen was low for all samples.

## Conclusion

The rising incidence of patients with CKD5 and the absence of clear data on survival benefit using the current haemodialysis regime calls for new and innovative approaches. Positive preliminary data suggests that adsorbent systems such as the one described may be used to augment high molecular weight and protein bound uraemic toxin removal, reducing chronic inflammatory stimulus and impacting the poor prognosis for patients with CKD5d.
